# Complex RNA metabolism in the chloroplast: an update on the *psbB* operon

**DOI:** 10.1007/s00425-012-1782-z

**Published:** 2012-10-13

**Authors:** Rhea Stoppel, Jörg Meurer

**Affiliations:** 1 Plant Molecular Biology (Botany), Department Biology I, Ludwig Maximilians University, Großhadernerstr. 2-4, 82152 Planegg-Martinsried, Germany; 2 Present Address: Joint BioEnergy Institute, Feedstocks Division, Lawrence Berkeley National Laboratory, 5885 Hollis St., Emeryville, CA 94608 USA

**Keywords:** Arabidopsis, Editing, Processing, Splicing, Stability

## Abstract

Expression of most plastid genes involves multiple post-transcriptional processing events, such as splicing, editing, and intercistronic processing. The latter involves the formation of mono-, di-, and multicistronic transcripts, which can further be regulated by differential stability and expression. The plastid pentacistronic *psbB* transcription unit has been well characterized in vascular plants. It encodes the subunits CP47 (*psbB*), T (*psbT*), and H (*psbH*) of photosystem II as well as cytochrome *b*_6_ (*petB*) and subunit IV (*petD*) of the cytochrome *b*_6_ *f* complex. Each of the *petB* and *petD* genes contains a group II intron, which is spliced during post-transcriptional modification. The small subunit of photosystem II, PsbN, is encoded in the intercistronic region between *psbH* and *psbT* but is transcribed in the opposite direction. Expression of the *psbB* gene cluster necessitates different processing events along with numerous newly evolved specificity factors conferring stability to many of the processed RNA transcripts, and thus exemplarily shows the complexity of RNA metabolism in the chloroplast.

## Introduction

The chloroplast evolved as a result of an endosymbiotic event in which a cyanobacterial ancestor was taken over by a eukaryotic cell. Though main parts of the original plastid genes were transferred into the nucleus, chloroplasts still have retained a separate genome. Chloroplast genes are embedded in the regulatory network of the cell enabling an adaptive and developmentally regulated chloroplast biogenesis, which is mainly controlled by nuclear factors (Stern et al. 2010). A highly sophisticated system of transcript maturation including endo- and exonucleolytic activities, splicing, editing, and modulation of RNA stability has been developed which is not exploited to the same extent in the free-living cyanobacterial ancestor. Various mechanisms can determine the stability of chloroplast mRNAs, including protection of RNA termini by proteins or RNA secondary structures. Since untranslated regions are not protected by ribosomes they are typical sites of rather unspecific endonucleolytic cleavage. Newly formed RNA termini are subject to fast digestion by exonucleases making accessibility to such sequences a key determinant of mRNA stability (Stoppel and Meurer 2012). Gene-specific transacting factors encoded in the nucleus can bind the 5′ UTR of mRNAs to protect them against 5′ → 3′ exonucleases (Drager et al. 1998), while the transcript 3′-end can in turn be stabilized by stable stem-loop structures or proteins, protecting from digestion by 3′ → 5′ exonucleases (Barkan 2011).

**Fig. 1:**
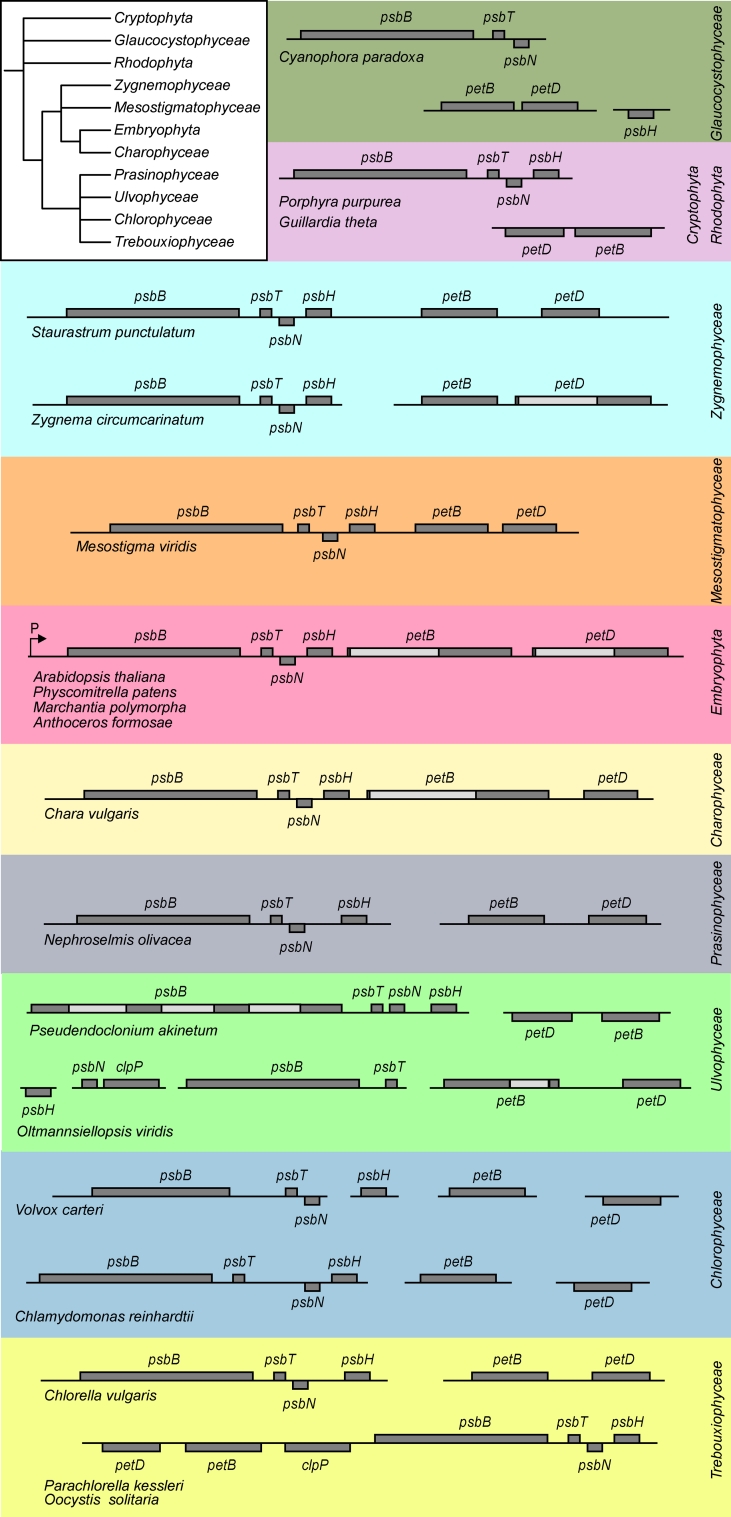
The *psbB* operon structure during evolution of the plant kingdom. The order of mono-, di-, and polycistrons of genes that are part of the *psbB* operon is shown for different organisms. A phylogenetic tree in the upper left corner shows the relationship among these organisms based on the nuclear genomes of the NCBI taxonomy tree (*iTOL.embl.de*). For details see text

**Fig. 2:**
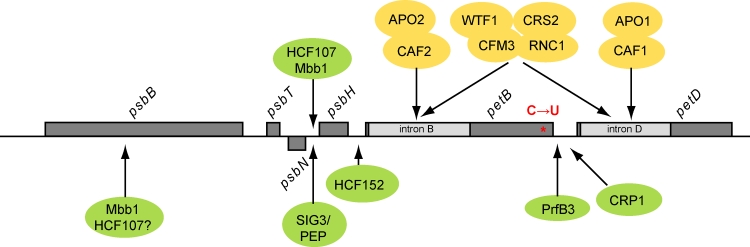
Complex RNA metabolism in the *psbB* operon. The structure of the *psbB* operon from *Embryophyta* is depicted in *grey*. The transcript stability factors HCF107, Mbb1, HCF152, PrfB3, CRP1 as well as the SIG3/PEP holoenzyme together with their corresponding/predicted binding sites are shown in *green*. Factors involved in splicing of the *petB* and *petD* introns are colored *yellow*. The *petB* editing site from maize and tobacco is drawn in *red*. The chronology of different events is still not clarified

Numerous nuclear-encoded factors have been acquired for processing and other post-transcriptional modifications of plastid transcripts (Stern et al. 2010; Barkan 2011). Most if not all protein-coding genes on vascular plant chloroplasts are found in polycistronic transcription units. Their intercistronic processing can differ between plant species and results in complex transcript pattern creating mono-, di-, and multicistronic transcripts which can further be regulated by differential stability. The *psbB*-*psbT*-*psbH*-*petB*-*petD* gene cluster has a promoter for the plastid-encoded RNA polymerase (PEP) and is highly conserved among vascular plants (Fig. 1). Each of the *petB* and *petD* genes contains a group II intron, which is spliced during post-transcriptional modification. Splicing along with intercistronic processing generates about 20 different mono-, di-, and oligocistronic transcripts (Barkan 1988; Westhoff and Herrmann 1988). Newly evolved specificity factors confer stability to many of these RNA transcripts by binding to their termini and blocking exoribonucleases. The evolution of the *psbB* cluster genes (Fig. 1), along with functions of the encoded proteins and known factors for transcript processing and stability events (Fig. 2) will be elaborated in this review.

## Evolution of the *psbB* operon

We analyzed the evolution of the *psbB* operon structure in representative sequenced plastid genomes of different plant species (Fig. 1). The conserved organization of the chloroplast *psbB* operon can be found in all vascular plants, the liverwort *Marchantia polymorpha*, the moss *Physcomitrella patens,* and the hornwort *Anthoceros formosae*. The zygnemophycean green algae *Staurastrum* and *Zygnema* have a similar organization of the *psbB* operon like the *Embryophyta* with only slight differences. Both belong to the *Charophyta* that are assumed to have given rise to land plants (Turmel et al. 2002). In *Staurastrum punctulatum* the genes are clustered in the same way as in vascular plants, however, both *petB* and *petD* do not carry introns, whereas in *Zygnema circumcarinatum* parts of the operon encoding the proteins of PSII and cytochrome *b*_6_ *f* complex are separated, and only *petD* contains an intron (Turmel et al. 2005). *Mesostigma viride*, also suggested to be a close relative of land plants (Lemieux et al. 2000; Karol et al. 2001), possesses complete *psbB* operons but is generally lacking introns. The charophycean green algae *Chara vulgaris* displays an almost typical *psbB* operon structure but lacks an intron in the *petD* gene.

In Chlorophyta the gene organization is different. *Chlorella vulgaris* and *Nephroselmis olivacea*, similar to *Zygnema*, have *psbB/T/N/H* and separate, uninterrupted *petB/D* clusters. However, it has to be noted that *Nephroselmis* similar to *Mesostigma* is lacking introns in its plastid genome. The other trebouxiophyceaen green algae *Parachlorella* and *Oocystis* have retained the conserved *psbB/T/N/H* organization but in contrast to *Chlorella* have the *petB* and *petD* genes moved 5′ of *clpP*, which in plastomes of vascular plants is located immediately upstream of *psbB*. *Chlamydomonas reinhardtii* and *Volvox carteri* have the vascular plant gene organization but *petB* and *petD* are not clustered and *petD* is transcribed on the opposite strand. *V. carteri* in addition has a separated *psbH*.

A quite different situation is found in two *ulvophyceaen* genomes. In *Pseudendoclonium akinetum* the *psbN* gene—with still unknown function—is on the same DNA strand as *psbB*, *psbT*, and *psbH* without changing the gene order, and *psbB* possesses three group I introns; the *petB/D* genes are clustered on the opposite strand and do not have introns. In *Oltmannsiellopsis viridis* the *psbB* cluster is completely fractured. While the dicistronic *psbN/clpP* and *psbB/T* are transcribed on the same strand followed by *petB* and *petD*, the *psbH* gene stands alone preceding all the other genes on the opposite strand. Again *petB* and *petD* are clustered and *petB* possesses a group IB intron, whereas *petD* has no intron (Pombert et al. 2006).

In Rhodophyta like f.e. *Porphyra purpurea* as well as in plastids of the secondary endosymbiont *Guillardia theta*, the *psbB/T/N/H* gene organization is conserved and *petB* and *petD* are clustered and intron-less, since plastomes of these species do not possess introns. In cyanelles of *Cyanophora paradoxa* in addition *psbH* is separated from *psbB/T/N*.

Therefore, it appears that during evolution, the pentacistronic *psbB*-*psbT*-*psbH*-*petB*-*petD* transcript with the *psbN* gene on the opposite DNA strand evolved as a result of fusing *psbB/T/N/H* and uninterrupted *petB/D* clusters after divergence of *Streptophyta* including *Charophyta* and land plants. The group II introns of *petB* and *petD* found in vascular plants might have been gained during evolution of the *Charophyta*. The diversity of the gene organization in *Chlorophyta* is consistent with the ability of introns to behave as mobile elements leading to intron gain and loss (Lambowitz and Belfort 1993).

The high degree of divergence as well as fluctuation of gene and intron composition of the *psbB* operon also attest the fast evolving operon organization accompanied by the recent acquisition of factors involved in processing of the primary transcript. In summary, the data indicate that the ontogenetic and phylogenetic integration of the chloroplast into the eukaryotic cell was predominantly established through controlling and functional clustering of plastid gene expression.

## Functions of *psbB* gene cluster encoded proteins

The *psbB* gene encodes the photosystem II (PSII) chlorophyll-binding protein of 47 kDa (CP47). Together with the chlorophyll-binding protein of 43 kDa (CP43) it builds up the inner light-harvesting complex (Barber et al. 1997). CP47 is closely attached to the PsbA/PsbD heterodimer and transfers excitation energy from the outer light-harvesting complexes onto them (Lucinski and Jackowski 2006).

Two small peptides, both associated with PSII, were originally designated PsbT: a 4 kDa protein encoded in the chloroplast (PsbTc) and an unrelated 11 kDa protein of nuclear origin (PsbTn) (Shi and Schröder 2004; Müh et al. 2008). PsbTc stabilizes the *Q*_B_ binding site in vivo that is essential for oxidation of reduced plastoquinone in darkness in an oxygen-dependent manner, possibly to keep the PSII acceptor site oxidized (Umate et al. 2008).

The PSII subunit H protein (PsbH) is important for PSII activity and was originally identified as an 8 kDa phosphoprotein in higher plant chloroplasts. The phosphorylation sites are thought to account for a regulatory role (Michel and Bennett 1987; Vener et al. 2001). Furthermore, PsbH might play a role in regulating PSII assembly/stability and repair of photodamaged PSII (Bennett 1977; Shi and Schröder 2004).

The small PSII subunit N (PsbN) is encoded on the opposite strand between *psbT* and *psbH.* *Synechocystis* mutants lacking both *psbH* and *psbN* showed no additional defects to *psbH* mutants alone, indicating that *psbN* is rather not essential for photoautotrophic growth (Mayes et al. 1993). In fact, the localization of PsbN as a PSII subunit has been a subject of a long debate that has not yet been satisfactorily solved. The gene product originally identified and named PsbN turned out to be PsbTc according to re-examinations of the PSII core oxygen-evolving complex (Kashino et al. 2002a). In addition, recent proteomics studies could not identify any PsbN associated to PSII (Gomez et al. 2002; Kashino et al. 2002b).

The last two genes of the *psbB* operon encode two proteins of the cytochrome *b*_6_ *f* complex. Having an oxidoreductase activity, this complex is one of the central points of electron transport through the thylakoid membrane (Allen 2002). In addition to cytochrome *b*_6_ (*petB*) and subunit IV (*petD*), this complex consists of cytochrome *f* (petA), the Rieske protein (*petC*), and the four small polypeptides PetG, PetL, PetM, and PetN (Schwenkert et al. 2007). Apart from its function in linear electron transport from PSII to PSI, the cytochrome *b*_6_ *f* complex is also involved in cyclic electron transport around PSI, regulation of gene expression, and reversible phosphorylation of plastid proteins (Joliot and Joliot 2006).

## Transcript specificity factors

### The sigma-like transcription factor SIG3

The activity of the PEP is regulated by sigma-like transcription initiation factors (SIG) that share a widely conserved C-terminal RNA polymerase sigma-70 factor domain. One of six SIG factors encoded in the Arabidopsis nuclear genome and with homologs only among *Embryophyta* is SIG3. The functionality of SIG3 is not essential for plastid functions and was proposed to depend on its attachment to thylakoid membranes (Privat et al. 2003). The strong reduction of *psbN* mRNA in *sig3* mutants as revealed by microarray and RNA gel blot analysis was proven to result from tight regulation of *psbN* gene expression by the SIG3-PEP holoenzyme binding to a promoter region upstream of *psbN* (Fig. 2) (Zghidi et al. 2007). Furthermore, *psbN* read-through transcription produces antisense RNA to *psbT* mRNA (Zghidi et al. 2007; Zghidi-Abouzid et al. 2011). It was shown that during photooxidative stress conditions the presence of this *psbT* antisense RNA leads to the formation of RNA double-strand hybrids and accordingly to translational inactivation of *psbT* (Zghidi-Abouzid et al. 2011). Thus, *psbT* mRNA can be protected from nucleolytic degradation by single-strand specific nucleases. Besides its function in transcription of *psbN* mRNA and *psbT* antisense RNA, SIG3 was recently shown to participate in transcription initiation of genes *atpI/H/F/A* of the large *atp* operon by specifically recognizing an internal promoter between *atpI* and *atpH* (Zghidi et al. 2007; Malik Ghulam et al. 2012).

### The *high*-*chlorophyll*-*fluorescence* phenotype protein HCF107

HCF107 is a tetratricopeptide repeat (TPR)-like protein with 11 half-a-TPR (HAT) helical repeats arranged in tandem (Sane et al. 2005; Hammani et al. 2012). Mutants of this gene are seedling lethal and therefore have to be maintained on sucrose-supplemented medium. Their inability to accumulate 5′-end processed *psbH* transcripts results in the loss of PsbH and consequently in the disruption of PSII activity (Felder et al. 2001; Sane et al. 2005). Accordingly, HCF107 was proposed to function in intercistronic processing or stabilization of the *psbH* 5′ UTR (Felder et al. 2001). It was suggested, that only those *psbH*-containing transcripts can be translated that have 5′ processed ends at position −45 with respect to the ATG start codon (Felder et al. 2001). Similar to the molecular function of the well-described PPR10 protein (Pfalz et al. 2009), processing at the −45 site would lead to unfolding of stable stem loops which otherwise would prevent translation. The HCF107 binding site was postulated to be at the *psbH* 5′-end as indicated by RNA footprint analysis with a small RNA defining the position of the processed *psbH* 5′-terminus by blocking 5′ → 3′ degradation (Zhelyazkova et al. 2012). This hypothesis was recently confirmed by RNA-binding studies, revealing that the sequence-specific RNA-binding properties of HCF107 come from the HAT motif (Fig. 2) (Hammani et al. 2012). Upon binding to its native RNA ligand in the *psbH* 5′ UTR, the local RNA structure undergoes conformational changes, which in turn protect the adjacent RNA from a 5′ → 3′ exonuclease in vitro, thus defining the 5′-end of processed *psbH* transcripts and stabilizing the downstream transcript. The *psbH* 5′ UTR and the translation initiation region are predicted to form stable duplexes if HCF107 is absent. Upon binding of HCF107, these inhibitory duplexes dissociate and expose the sequence so that ribosomes can easily bind, resulting in increased *psbH* translation efficiency (Hammani et al. 2012).

In a similar manner, HCF107 could be involved in translation of the *psbB* gene, since along with PsbH also the CP47 protein (encoded by the *psbB gene*) was reported to be missing in *hcf107* mutants. On the other hand, there are no sequence similarities between the 5′ *psbH* and *psbB* sequences. Since it was reported that *hcf107* mutants grown under very low light are able to accumulate slight amounts of CP47 (Plücken et al. 2002), the translational deficiencies are likely to represent a secondary effect of the missing PsbH rather than representing a dual function of HCF107.

### The *psbB* mRNA maturation factor Mbb1

The well-characterized *Chlamydomonas* protein Mbb1 is sharing a sequence identity of about 40 % to the Arabidopsis HCF107 and similar proteins also occur in other *Chlorophyta* species. Knockout mutants of *mbb1* are affected in *psbB* 5′-end processing and *psbH* processing/stability and predominantly fail to accumulate the *psbB* encoded CP47 (Fig. 2) (Vaistij et al. 2000a). This is inconsistent with the Arabidopsis *hcf107* mutation, that is only affecting *psbH* accumulation. *Chlamydomonas* *mbb1* mutants consequently display broader defects in PSII complex assembly (Monod et al. 1992). Furthermore, Mbb1 is a stromal protein compared to HCF107 being a membrane bound protein, most likely because the similarities between both proteins are spanning only the TPR region. Despite these differences phylogenetic analysis has clearly shown that both proteins are evolutionary orthologs (Felder et al. 2001). Similar to HCF107, Mbb1 has ten HAT motifs arranged in tandem. These motifs most likely mediate protein–protein interaction, supported by the fact that Mbb1 has been identified as part of a 300 kDa complex (Vaistij et al. 2000b).

### The *high*-*chlorophyll*-*fluorescence* phenotype protein HCF152

The Arabidopsis protein HCF152 is a member of the pentatricopeptide repeat (PPR) protein family and forms homodimers via its C-terminal non-PPR regions (Nakamura et al. 2003). It is required for the accumulation of 5′ or 3′ processed RNA termini mapping in the intercistronic region of *psbH*-*petB* in *Arabidopsis* chloroplasts and accordingly *hcf152* mutants are lacking the cytochrome *b*_6_ *f* complex (Meierhoff et al. 2003; Nakamura et al. 2003). It has been shown that the *psbH* 3′-end maps downstream of the *petB* 5′-end with an overlap of about 25-nt (Pfalz et al. 2009). After initial uncertainties about the HCF152 binding site, it has now been clearly shown, that this 25-nt overlap in the *psbH*–*petB* intergenic region constitutes the binding site for HCF152 in analogy to the recently characterized PPR10 and HCF107 proteins (Fig. 2). Thus, HCF152 is an another example for a transcript specificity factor, that defines processed transcript termini and protects upstream and downstream RNA transcripts from digestion by 5′ → 3′ or 3′ → 5′-exonucleases (Zhelyazkova et al. 2012). Similar to HCF107 homologies to proteins from other organisms are restricted to *Embryophyta* and *Chlorophyta*, clearly showing the recent evolvement of these stability factors.

### The ribosomal peptide chain release factor B (PrfB)-like protein PrfB3

The protein PrfB3 is localized in the chloroplast stroma in a *petB* RNA-containing complex (Stoppel et al. 2011). Absence of the PrfB3 gene in sequenced genomes of cyanobacteria, red, green, and diatom algae suggests that PrfB3 evolved after the divergence of vascular plants, probably as a result of a duplication of the ancestral PrfB gene and subsequent loss of the peptide chain release function, followed by loss of the two conserved motifs, harboring the sites for UGA stop-codon recognition and peptidyl-tRNA hydrolysis. It is tempting to suggest that PrfB3 might have arisen after the appearance of the typical *psbB* operon organization for higher plants. This hypothesis can be further substantiated by absence of PrfB3 in *C.* *reinhardtii* which does not involve *petB* and *petD* as part of the *psbB* transcription unit. PrfB3 is essentially required for photoautotrophic growth and mutations in this gene lead to a specific deficiency of the cytochrome *b*_6_ *f* complex (Stoppel et al. 2011). PrfB3 has been shown to bind specifically to the 3′ region of processed *petB* transcripts, stabilizing, and protecting them from digestion by exonucleases (Fig. 2). Furthermore, the stability of these transcripts is regulated in a light- and stress-dependent manner, to adjust cytochrome *b*_6_ levels (Stoppel et al. 2011). Thereby, overall photosynthesis rates can be controlled according to the plants’ needs. Interestingly, no RNA footprint has been identified for the 3′ *petB* region, indicating that non-PPR proteins like PrfB3 underlie a different mechanism of RNA stabilization than PPR proteins, possibly by binding to the transcript in a less strong manner. This would also facilitate faster and more sensitive regulation of the transcript’s RNA stability.

### The chloroplast RNA processing 1 (CRP1) protein

Originally, the PPR protein CRP1 had been described to be required for the accumulation of processed 5′- and 3′-termini in the maize *petB*–*petD* intergenic region (Fig. 2) (Barkan et al. 1994; Fisk et al. 1999). *crp1* mutants lack both monocistronic *petB* and *petD*, but in contrast to *prfB3* are able to accumulate cytochrome *b*_6_ protein to normal levels (Barkan et al. 1994). Accordingly, the 3′ *petB* and 5′ *petD* transcript ends of Arabidopsis do not overlap—whereas in maize they do—and independent intercistronic processing events produce the respective ends (Barkan et al. 1994; Stoppel et al. 2011). Thus, similar to *prfB3*, the lack of a monocistronic transcript can lead to severe defects, indicating that normal levels of a polycistronic precursor transcript are not always sufficient for translation of a protein. A reason for the inability to translate polycistronic transcripts can be the formation of stable hairpins that inhibit the start codon from being recognized by the translation machinery (Barkan et al. 1994). The necessity of such a regulatory mechanism is evolutionary young demonstrated by the fact that again homologs to CRP1 can only be found among *Embryophyta* and some *Chlorophyta*. In addition to the lack of monocistronic *petB* and *petD*, *crp1* mutants display defects in translation of *petA,* another subunit of the cytochrome *b*_6_ *f* complex, and *psaC*, a PSI subunit (Fisk et al. 1999). However, it seems that both defects occur independent from each other and binding affinity of a recombinant CRP1 protein has been shown only for *petA* transcripts (Williams-Carrier et al. 2008).

## Splice factors of the *psbB* operon

The *psbB* operon of higher plants has two group II intron-containing genes, *petB* and *petD*. Though being derived from ‘self-splicing’ ribozymes, introns of higher plant chloroplasts depend on specific splice-factors for proper intron folding into catalytically active structures (Barkan 2011). The complexity of protein association to group II introns involves for example six proteins being required for splicing of *petB* and *petD*, respectively. This includes genes of the APO domain family (APO1 and APO2), the CRM domain family (CAF1, CAF2, CRS2, CFM3), and the proteins WTF1 and RNC1 (Fig. 2). Except CRS2 that also has homologs among *Chlorophyta* all other splice factors can only be found in *Embryophyta*, consistent with the sole presence of both introns in this clade. Furthermore, this is an evidence that both intron splice sites and corresponding factors depend at least in part on each other.

The mutant *apo1* was originally described as being affected in PSI assembly (Amann et al. 2004) and APO1 protein was later found to be part of ribonucleoprotein particles involved in splicing of group II intron transcripts. While the major function of APO1 is in splicing of the second intron of *ycf3*, *apo1* mutants also fail to properly splice *petD* and *clpP*-intron 1 (Watkins et al. 2011). Similarly, APO2 seems to account for splicing of the *petB* intron (Barkan 2011). CAF1, CAF2, CRS2, and CFM3 are all members of the chloroplast RNA splicing and maturation (CRM) domain family (Asakura et al. 2008). The paralogs CAF1 and CAF2 build a heterodimeric complex that functions together with CRS2, a peptidyl-tRNA hydrolase homolog (Ostheimer et al. 2003, 2006). CFM3 associates with the CRS2/CAF complex to promote splicing of a certain set of group II introns, including those of *petB* and *petD* (Asakura et al. 2008). Knockout mutants of either of these genes exhibit strong splicing defects, indicating the non-redundancy of the respective proteins. Other members of the CRM domain family have been shown to enhance intron folding and this was also suggested to be the function of CFM3 (Ostersetzer et al. 2005; Asakura et al. 2008). In contrast, the role of CAF1 and CAF2 seems to be restricted to the recruitment of the splicing factor CRS2 to specific introns. The exact role of CRS2 however still has to be elucidated. The protein ‘What’s This Factor 1’ (WTF1) and an RNase III domain protein (RNC1), that does not exhibit endonucleolytic activity, were independently discovered through co-immunoprecipitation analyses with proteins of the CRM domain family (Watkins et al. 2007; Kroeger et al. 2009). In a similar manner to the CRM proteins, WTF1 and RNC1 build a heterodimer that binds RNA and associates with several group II introns, among them the *petB* and *petD* introns (Kroeger et al. 2009). The reason for this highly complex RNA splicing machinery for group II introns is still more than cryptic and future studies will have to show specific functions for each of the proteins described.

## Editing in the *psbB* operon

During post-transcriptional RNA editing events the exchange of individual nucleotides in transcripts, altering amino acid identity or creating new translation initiation codons or stop codons, is often essentially required for the production of functional proteins. While editing in land plants is usually a cytidine-to-uridine (C-to-U) change some moss and fern organelles have additional U-to-C editing reactions (Castandet and Araya 2011). A recent study of the moss *Takakia lepidozioides* identified 116 C-to-U conversions in transcripts of the *psbB* operon (Sugita et al. 2006).

One of these editing sites at position 204 within the *petB* coding region is conserved in tobacco and maize (Fig. 2) but not in Arabidopsis or *Chlamydomonas* (Freyer et al. 1993; Tillich et al. 2005; Zito et al. 1997). This editing event changes the amino acid proline at this position into leucine and already occurs before splicing and processing of the *psbB* operon primary transcript and is therefore an independent processing step in the maturation of the *psbB* transcription unit. All factors involved in editing identified so far are members of the PPR protein family (Bentolila et al. 2012; Fujii and Small 2011). One possibility to identify the factor for this specific editing site could be to look for PPR proteins present in tobacco and maize but not in species where this editing site is not found.

## Conclusions/perspectives

It appears that plastid gene expression is mainly regulated and controlled by products of newly evolved nuclear genes or by conserved proteins, which often acquired new functions and/or new domains. Extensive endonucleolytic cleavage events were important to extract individual gene segments from the polycistronic context and to independently regulate both stability and translation of each gene, irrespective whether they are co-transcribed or not. The specificity by which the expression of plastid genes is regulated is evident by the variety of observed mutant phenotypes. Another example is the higher divergence of target sequence elements in UTRs and intergenic regions as compared to conserved coding regions even between closely related species (Greiner et al. 2008a, b). Coding regions are assumed not to be significantly subjected to the control of gene expression. This is also consistent with the fact that different genome-plastome incompatibilities are based on malfunction of plastid gene expression. Importantly, the frequent occurrence of novel plant-specific genes required for the chloroplast mRNA homeostasis demonstrates that transcript regulation represents a fast evolving process during evolution. In contrast, nuclear-encoded factors such as HCF136, ALB3, VIPP1, YCF3, PSB27, and HCF101 required for assembly of conserved structures like the photosynthetic complexes mainly remained conserved and have already been established in cyanobacteria.

## Abbreviations

PSII: Photosystem II
PEP: Plastid-encoded RNA polymerase
PPR: Pentatricopeptide repeat
TPR: Tetratricopeptide repeat
HAT: Half-a-TPR
